# Development of Conductometric Sensor Based on 25,27-Di-(5-thio-octyloxy)calix[4]arene-crown-6 for Determination of Ammonium

**DOI:** 10.1186/s11671-016-1317-9

**Published:** 2016-02-25

**Authors:** O. Y. Saiapina, S. G. Kharchenko, S. G. Vishnevskii, V. M. Pyeshkova, V. I. Kalchenko, S. V. Dzyadevych

**Affiliations:** Laboratory of Biomolecular Electronics, Institute of Molecular Biology and Genetics of National Academy of Sciences of Ukraine, 150 Zabolotnogo Str., 03680 Kyiv, Ukraine; Institute of Organic Chemistry of National Academy of Sciences of Ukraine, 5 Murmanska Str., 02660 Kyiv, Ukraine

**Keywords:** Ammonium, 25,27-di-(5-thio-octyloxy)calix[4]arene-crown-6, Conductometric transducers, Ion selectivity, Analytical characteristics

## Abstract

The conductometric sensor based on 25,27-di-(5-thio-octyloxy)calix[4]arene-crown-6 was developed for the quantitative analysis of ammonium. The calixarene was immobilized on the surface of the planar interdigitated electrodes by attachment of its dialkyl sulfide groups to the surface of the gold electrodes. The intrinsic ability of the calixarene to capture ammonium was studied in the conductometric measuring mode and by the electrochemical impedance spectroscopy. The developed sensor showed high selectivity to ammonium in the presence of mono-, di-, and trivalent cations. Selective and highly sensitive detection of ammonium resulted from the complexation between the ammonium ions and a crown-ether fragment of the upper rim of the 25,27-di-(5-thio-octyloxy)calix[4]arene-crown-6 macrocycle. The developed sensor had high signal repeatability. Its sensitivity was found to be satisfactory for the forthcoming sensor application in the water-sample analysis; the linear range was 0.01–1.5 mM and limit of detection 10 μM.

**Electronic supplementary material:**

The online version of this article (doi:10.1186/s11671-016-1317-9) contains supplementary material, which is available to authorized users.

## Background

Nowadays, an increase in the ammonium concentrations in the surface water systems causes a significant environmental and economic concern. The possible sources of ammonium and ammonia in surface water systems are land-applied manure and biosolids, fertilizer spills, septic systems, raw sewage, snow, rainfall, animal feedlot runoff, surface runoff into tile inlets, eroded soil and sediment, airborne ammonia, direct deposit by aquatic organisms, wildlife manure, land-applied fertilizer (ammonium containing) for crop and turf production, fertilizer on sidewalks and driveways, manure storage structures, manure stockpiles, fertilizer facilities, decay of aquatic organisms and organic materials in water, etc. [1, 2]. In the water systems, two nitrogen forms coexist in a certain ratio depending on the water pH, temperature, and ionic strength—unionized NH_3_ (ammonia) and ionized NH_4_^+^ (ammonium). Since a direct reaction between ammonia and water produces ammonium (Eq. (1)), the ammonia analysis in aqueous media often comes to the detection of ammonium ion concentration.

1 $$ {\mathrm{NH}}_3 + {\mathrm{H}}_2\mathrm{O}\ \leftrightarrow\ {\mathrm{NH}}_4 + {\mathrm{OH}}^{-} $$

New analytical tools based on the chemical entities capable of inducing selective binding and transport of ionic or neutral species are of growing interest nowadays. Amongst the synthetic receptors widely applied in the sensor development (e.g., crown ethers, cyclophanes, cyclodextrins), calixarenes occupy a pivotal position. Being the phenolic [1]_n_metacyclophanes obtained by the high-precision cyclo-condensation of the *p*-substituted phenols with formaldehyde, calix[n]arenes possess intramolecular lipophilic cavities formed by aromatic rings of the macrocyclic skeleton. The structural diversity of calix[n]arenes is stipulated by their conformational isomerism and is caused by the hampered rotation of the phenolic fragments around the Ar–CH_2_–Ar bonds. In the case of calix[4]arenes, such a rotation is hampered by the bulky substituents near to the atoms of oxygen belonging to a lower rim of the macrocycle [3].

To date, the methods of synthesis of calix[4]arene derivatives in the stereochemically rigid conformations—*cone*, *partial cone*, *1,2-alternate*, and *1,3-alternate*—have been developed. They show unique ability to recognize ions and molecules by the host-guest principle owing to a variety of the noncovalent supramolecular interactions (hydrogen bonds, ion-dipole, cation-π, anion-π, CH-π, stacking, van der Waals interactions, etc.). These properties open wide perspectives of practical application of the calix[4]arenes in different branches of chemistry, physics, biology, medicine, and sensor technologies.

It is well-known that the recognition of ammonium cations is characteristic for 18-crown-6 ether and its derivatives that form stable “host-guest” complexes through a system of cooperative ion-dipole and hydrogen bonds of ammonium cation with oxygen atoms of crown ether [4]. Thus, the combination of structural fragments of calixarenes and crown ether in one molecule allows achieving high efficiency and selectivity of ammonium cation binding.

These properties can be observed in the molecule of calix[4]arene-18-crown-6 in the *1,3-alternate* conformation that exhibits the complementarity of a stereochemically rigid three-dimensional molecular cavity to the tetrahedral ammonium cation [5, 6]. The lipophilic benzene rings of the cavity protect the ammonium cation against hydration, thus enhancing the ion-dipole and hydrogen bonds in the supramolecular “host-guest” complex.

As we showed it earlier, ammonium can be quantitatively determined with natural zeolite immobilized on the surface of the conductometric interdigitated transducers [7]. Here, we aim at developing a novel ammonium-selective sensor based on 25,27-di-(5-thio-octyloxy)calix[4]arene-crown-6, that is calix[4]arene-18-crown-6 in the *1,3-alternate* conformation, synthesized by us for the first time, and studying the analytical characteristics of the developed sensor targeting to its further application in a real sample analysis. In our previous work, we have already reported the use of clinoptilolite for ammonium analysis with the conductometric sensor [7]. However, the selectivity of the sensor mentioned was not sufficient for the sensor application in the complex media. In the work presented, we continue to search for the materials suitable for highly sensitive and selective recognition of ammonium. For this purpose, 25,27-di-(5-thio-octyloxy)calix[4]arene-crown-6 was designed and applied to study its potentiality to be used for gold functionalization and ammonium capture with high selectivity and sensitivity at once.

## Methods

### Chemicals

The reagents used were the following: ammonium nitrate, sodium nitrate, potassium nitrate, magnesium nitrate, calcium nitrate tetrahydrate, aluminum nitrate nonahydrate, sulfuric acid (conc. 95–98 %), hydrogen peroxide solution (conc. 35 %), and toluene supplied by Macrochem (Ukraine). A phosphate buffer solution was prepared from potassium nitrate monobasic and sodium phosphate dibasic (Helicon, Russian Federation); its concentration was 5 mmol L^−1^ and pH 6.1–6.2 unless stated otherwise. The chemicals were at least of purified grade. All solutions used were prepared using ultrapure water (the water was obtained from a Millipore purification system; its resistivity was no less than 18.2 MΩ cm).

### Synthesis of 25,27-Di-(5-thio-octyloxy)calix[4]arene-crown-6

**Fig. 1:**
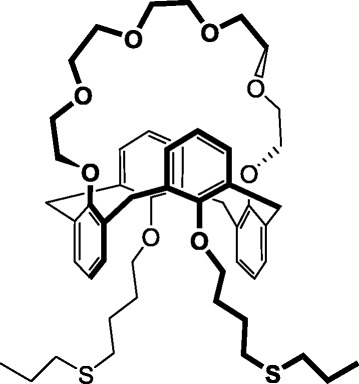
Structural formulae of 25,27-di-(5-thio-octyloxy)calix[4]arene-crown-6

The key component of the sensor membrane, 25,27-di-(5-thio-octyloxy)calix[4]arene-crown-6 (Fig. 1), was synthesized as follows. In the first stage, tetrahydroxycalyx[4]arene was alkylated by 1,4-dichlorobutane in the presence of potassium carbonate as a base, which resulted in the formation of 25,27-di-(4-chlorobutoxy)calix[4]arene in the *cone* conformation. In the second stage, the reaction of cyclization of di-(4-chlorobutoxy)calix[4]arene with ditosylate pentaethylene glycol in the presence of cesium carbonate resulted in obtaining 25,27-di-(4-chlorobutoxy)calix[4]arene-crown-6 in the *1,3-alternate* conformation. In the final stage, the chlorine atoms of 25,27-di-(4-chlorobutoxy)calix[4]arene-crown-6 were substituted with propyl sulfide groups by the reaction of 25,27-di-(4-chlorobutoxy)calix[4]arene-crown-6 with propylmercaptan in the solution of tetrahydrofuran in the presence of sodium hydride.

For more details on the synthesis of 25,27-di-(5-thio-octyloxy)calix[4]arene-crown-6 (further may be simply mentioned as “calixarene”) and its characterization, please refer to Additional file 1.

### Conductometric Transducers

**Fig. 2:**
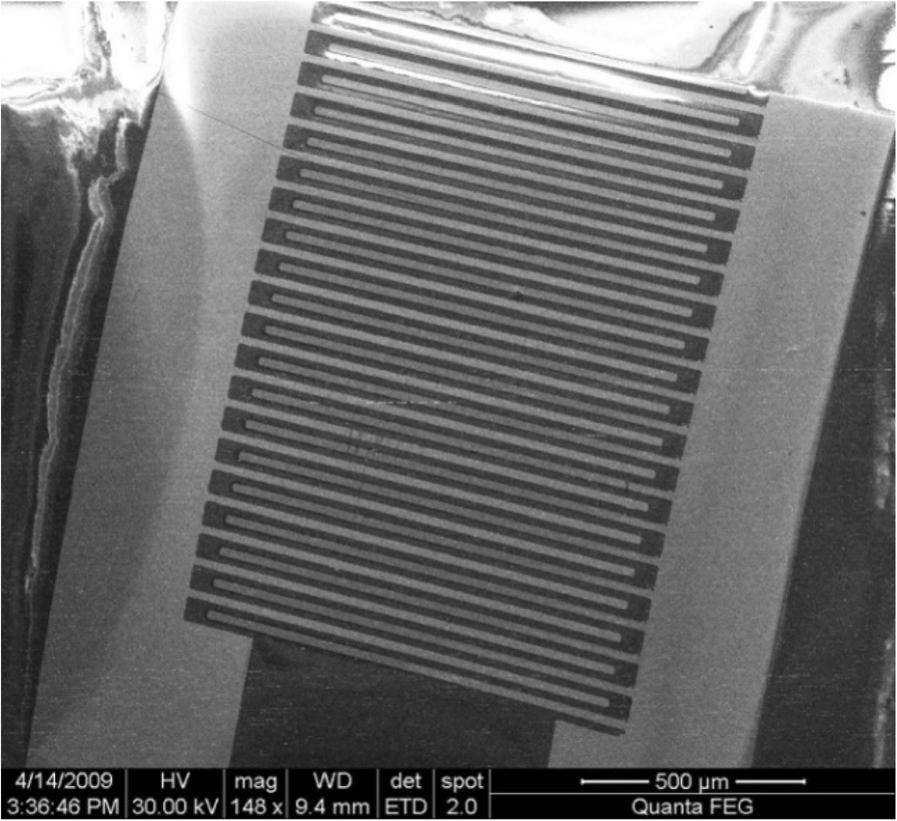
General view of two differential conductometric transducers embedded in the holders

**Fig. 3:**
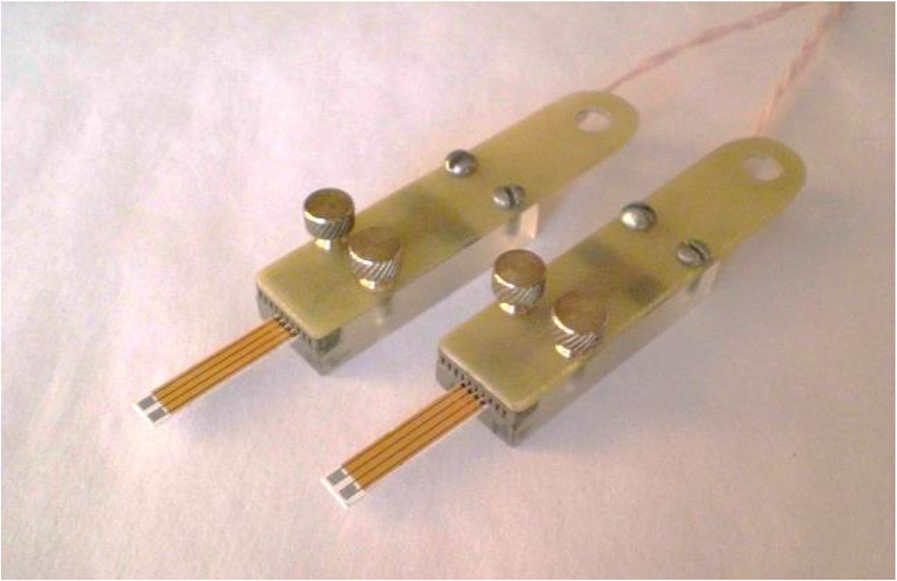
Layout of the sensitive area of one pair of interdigitated electrodes of a gold conductometric transducer. SEM image obtained using “FEI Quanta 400F”

The conductometric transducers were 5 × 30 mm in size, and each of them consisted of two identical pairs of gold interdigitated electrodes deposited onto a ceramic support. The sensitive area of each electrode pair was about 2.9 mm^2^. Both the digit width and interdigital distance were 10 μm, and their length was ~1.5 mm. Conductive buses of the electrodes were covered with insulating material, except for the active region and the contact zone. The usage of two electrode pairs enabled differential mode of measurements. The image of a pair of gold interdigitated electrodes obtained by scanning electron microscopy and the overall view of the conductometric transducers fixed in the holders are presented in Figs. 2 and 3, respectively.

The transducers were manufactured at the V.Ye. Lashkaryov Institute of Semiconductor Physics of National Academy of Sciences of Ukraine (Kyiv, Ukraine). The electrodes were fabricated by thermo-vacuum sputtering of chromium (5 nm) and gold (150 nm) onto a non-conducting ceramic substrate. The chromium served to improve gold adhesion to the substrate in the electrodes fabrication.

Prior to deposition of any selective material on the transducers' sensitive areas, they were treated with piranha solution (consisted of H_2_SO_4_ (conc. 99.9 %) and H_2_O_2_ (conc. 35 %) in ratio of 2.34:1) then thoroughly rinsed in Milli Q water and treated with absolute ethanol. For the sensor fabrication, calixarene was deposited on the sensitive area of one pair of electrodes which was designated as a working membrane (for the information on the working membrane preparation, please refer to 2.5). The second pair of electrodes was not modified and was applied as a reference.

### Preparation of the Working Membrane of the Calixarene-Based Sensor

**Fig. 4:**
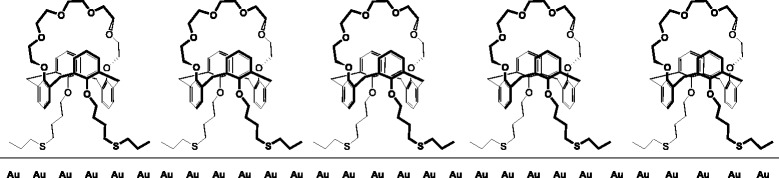
Schematic representation of atomic surface of gold covered with a monolayer of 25,27-di-(5-thio-octyloxy)calix[4]arene-crown-6

The structural features of the calixarene compound were considered as a base for its successful immobilization as well as in the ammonium detection using a conductometric measuring mode. These features were as follows. The upper rim of 25,27-di-(5-thio-octyloxy)calix[4]arene-crown-6 (Fig. 1) contained the structural fragment of the crown ether capable of the selective complexation with cations of the alkali metals; the lower rim contained the dialkyl sulfide groups capable of chemical binding to the surface of atomic gold (Fig. 4).

The working membrane was prepared in the following way. 25,27-Di-(5-thio-octyloxy)calix[4]arene-crown-6 obtained in the preceding stage was further mixed with toluene in the ratio of 1:1; here, the homogeneous “calixarene-toluene” mixture was prepared in the argon atmosphere to prevent the 25,27-di-(5-thio-octyloxy)calix[4]arene-crown-6 oxidation. Before modification of electrodes, their sensitive surfaces, i.e., both pairs of gold interdigitated electrodes, were kept for 1–2 min in a freshly prepared solution based on hydrogen peroxide and sulfuric acid (in the ratio of 2.34:1). Afterwards, the entire sensitive surface was washed in Milli Q water, air-dried, and treated with a concentrated solution of ethyl alcohol.

Further modification of gold with 25,27-di-(5-thio-octyloxy)calix[4]arene-crown-6 was performed for one pair of electrodes only. For this, 0.5 μL of the “calixarene-toluene” mixture obtained in the preceding stage were deposited as a thin layer on one pair of electrodes using a micropipette. The procedure was performed at room temperature. Afterwards, the electrodes were kept in the dark for 60 min to undergo the process of self-assembly of the monolayer from the 25,27-di-(5-thio-octyloxy)calix[4]arene-crown-6 molecules. The procedure was carried out in triplicate, with 60-min intervals between the depositions. After the last deposition, the sensor was kept at room temperature for 60 min and then left dry in the environment at +4 °C for 24 h to complete the formation of ordered molecular layer of 25,27-di-(5-thio-octyloxy)calix[4]arene-crown-6 on the electrode surface. In the final stage, the sensor was washed for 30 min with fresh portions of 5 mM KH_2_PO4–Na_2_HPO_4_ buffer at constant stirring.

### Electrochemical Equipment for Conductometric Measurements

**Fig. 5:**
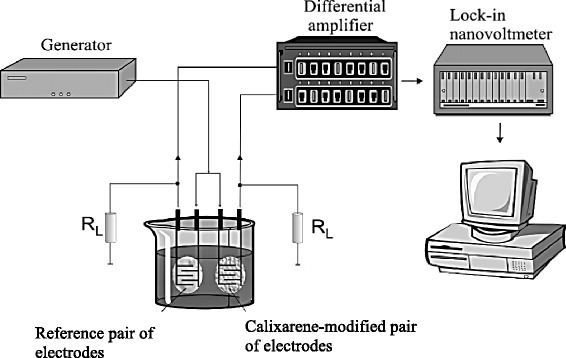
Scheme of conductometric set-up (reprinted from S.V. Dzyadevych and A.P. Soldatkin [12])

The conductometric measurements of ammonium with the prepared sensor were carried out in both differential and one-channel measuring modes. The differential mode was performed using the stationary experimental set-up [8, 9] that consisted of the elements schematically presented in Fig. 5. An alternative voltage (frequency of 100 kHz and amplitude of 10 mV) from the low-frequency signal generator “G3-118” (Ukraine) was applied to two pairs of interdigitated electrodes placed in the measuring cell. The circuit load resistance was *R*_*L*_ = 1 kΩ. The electrode output signals entered the differential amplifier “Unipan-233-6” (Poland). The obtained differential signal entered the selective lock-in nanovoltmeter “Unipan-232B” (Poland) where a real component of the analytical significance (i.e., conductivity) was selected out of the total output signal and recorded in the appropriate way. The measuring cell used was of 3 mL in volume, and magnetic stirring was applied throughout the measurements.

The electrochemical impedance spectroscopy (EIS) studies were performed with “VoltaLab 80” (model PGZ 301, Radiometer Analytical, France). Measurements were carried out in a frequency range of 100 mHz to 100 kHz and amplitude of 10 mV in a two-electrode configuration.

## Results and Discussion

### Selectivity of the 25,27-Di-(5-thio-octyloxy)calix[4]arene-crown-6-Based Sensor

The most essential characteristic of the ion-selective sensor is its response to the target ion in the presence of other ions. Usually, a term “selectivity coefficient” is well-known in regard to the potentiometric ion-selective electrodes [10] and defines an ability of an ion-selective electrode to distinguish a certain ion among the variety of other ions in the same solution. As for the conductometric sensors, the conductometric selectivity coefficient can be determined by fixed interference method (FIM) by Eq. (2) reported by Cammann [11], as the definition of *K*^Con^ was shown by him to be analogous to *K*^Pot^.

2 $$ {K}_{A,B}^{\mathrm{Con}} = \raisebox{1ex}{${a}_A$}\!\left/ \!\raisebox{-1ex}{${a}_B^{Z_A/{Z}_B}$}\right., $$

where *a*_*A*_ is the activity of a primary ion in the solution containing an interfering ion of the constant activity, *a*_*B*_ is the activity of an interfering ion, and *Z*_*A*_ and *Z*_*B*_ are the charges of primary and interfering ions, respectively.

**Fig. 6:**
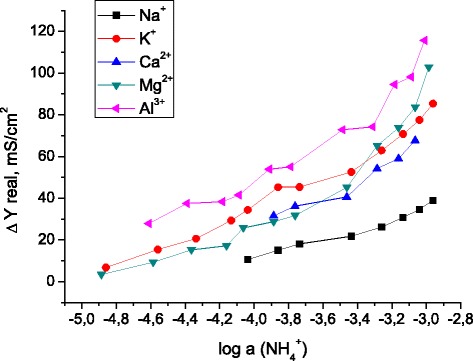
Dependence of admittance on ammonium activity found for the calixarene-based membrane of the sensor (EIS in the aqueous solutions of NaNO_3_, KNO_3_, Ca(NO_3_)_2_, Mg(NO_3_)_2_, and Al(NO_3_)_3_)

The responses to ammonium were obtained for the 25,27-di-(5-thio-octyloxy)calix[4]arene-crown-6-based membrane in the aqueous solutions of NaNO_3_, KNO_3_, Ca(NO_3_)_2_, Mg(NO_3_)_2_, and Al(NO_3_)_3_ (the concentration of each solution was 5 mM). Using the FIM method, the dependences of admittance on ammonium concentration in each solution were studied (Fig. 6), and the conductometric selectivity coefficients of the calixarene-based membrane determined by Eq. (2) were summarized in Table 1.

**Table 1:** Selectivity coefficient values of membrane based on 25,27-di-(5-thio-octyloxy)calix[4]arene-crown-6 for ammonium determination

Interfering ion (*B*)	Selectivity coefficient, ${K}_{\mathrm{NH}4,\ B}^{\mathrm{Con}}$	Logarithm of selectivity coefficient, log ${K}_{\mathrm{NH}4,\ B}^{\mathrm{Con}}$
Na^+^	3.8 × 10^−1^	−0.923
K^+^	2.8 × 10^−1^	-1.095
Mg^2+^	9.8 × 10^−2^	-2.243
Ca^2+^	1.2 × 10^−1^	-2.249
Al^3+^	7.3 × 10^−2^	-2.458

As seen from Table 1, the calixarene-based membrane demonstrated high selectivity toward NH_4_^+^ as compared to other ions such as sodium, potassium, calcium, magnesium, and aluminum that are essential components of the water samples. Comparison of the selectivity coefficients of the calixarene-based membrane with those of the clinoptilolite-based membrane obtained by the authors earlier [7] showed that the calixarene sample applied had better discrimination ability toward ammonium, giving promise for its further use.

### Analytical Characteristics of the 25,27-Di-(5-thio-octyloxy)calix[4]arene-crown-6-Based Sensor

Conductivity variations in the pre-electrode area, containing calixarene, in response to ammonium injections were studied in the cation-free medium (ultrapure water) in the differential mode of measurements.

**Fig. 7:**
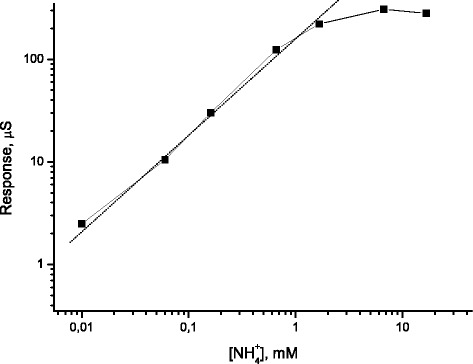
Calibration curve of the conductometric sensor based on 25,27-di-(5-thio-octyloxy)calix[4]arene-crown-6 for ammonium obtained in the differential mode of measurements (measurements in the ultrapure water)

The conductometric responses to ammonium of the calixarene-based sensor (Fig. 7) obtained in a wide concentration range demonstrated the analytical performance of 25,27-di-(5-thio-octyloxy)calix[4]arene-crown-6 in the range of 0.01–10 mM ammonium. The linear concentration range was found to be 0.01–1.5 mM, and the sensor sensitivity was about 8.2 μs mM^−1^ (RSD, 2–5 %, *n* = 3).

Comparison of signals of a bare electrode of a sensor and that of a calixarene-based membrane revealed that the differential signal of a sensor resulted from the conductivity increase just in the calixarene-based membrane, whereas the bare electrode did not record the conductivity variations drastically in the same solution (it measured the bulk conductivity of solution under the increase of ammonium concentration in it). This observation gives evidences for the applicability of toluene as a solvent for calixarene immobilization as well. It allows us to presume that toluene could provide a strong interaction between calixarene and gold surface (Au–S binding) and act positively making the calixarene molecules accessible enough to the guest ions (as a calixarene sample is of lipophilic nature). At the same time, selective and highly sensitive detection of ammonium resulted from the complexation between ammonium ions and a crown-ether fragment of the upper rim of the 25,27-di-(5-thio-octyloxy)calix[4]arene-crown-6 macrocycle provoking the conductivity growth in the calixarene/solution layer adjacent to the electrode.

**Fig. 8:**
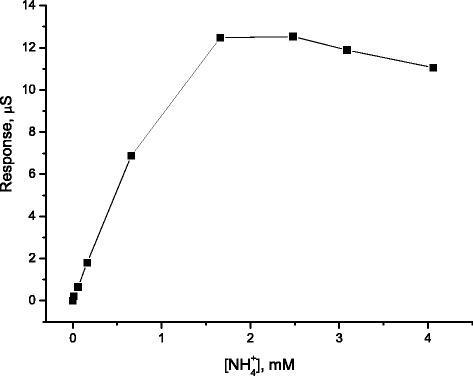
Calibration curve of the conductometric sensor based on 25,27-di-(5-thio-octyloxy)calix[4]arene-crown-6 for ammonium obtained in the differential mode of measurements (measurements in 5 mM KH_2_PO_4_–Na_2_HPO_4_ buffer, pH 6.2)

To investigate the analytical performance of the calixarene-based sensor in the presence of interfering ions, its calibration curve in a phosphate buffer solution (5 mM KH_2_PO4–Na_2_HPO_4_, pH 6.2) was obtained (Fig. 8).

As seen in Fig. 8, the calixarene-based membrane had a much lower amplitude of responses to ammonium if compared with the analog responses obtained in the ultrapure water (Fig. 7). In addition, the sensor saturation in the buffer solution was observed near to 2 mM ammonium (Fig. 8) in contrast to 10 mM as detected in the cation-free medium (Fig. 7). This last experiment suggests us to pay a considerable amount of attention to the working solution parameters for the further analysis of a real sample when using the developed sensor. However, optimization of the working solution composition is usually required for the adequate performance of any ion-selective electrode or ion-selective sensor.

### Response Time, Stability, and Potentiality for the Real Sample Analysis

As a response time of the sensor was taken the time required to achieve 95 % of the conductometric response (at the steady-state equilibrium) to 1 mM ammonium. Its value was found to be 5–10 s and did not change regardless of the way in which the conductivity shifts were recorded — from low to high concentrations or *vice versa*.

**Fig. 9:**
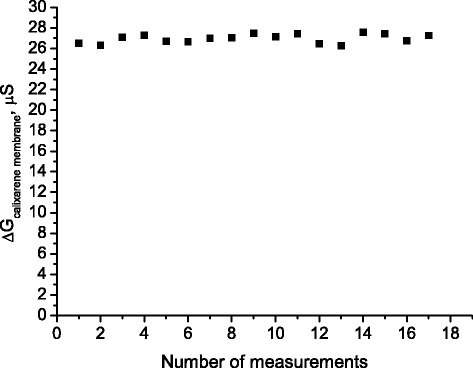
Signal repeatability of the sensor based on 25,27-di-(5-thio-octyloxy)calix[4]arene-crown-6 for ammonium determination. Responses to 5 mM ammonium

The structural and spatial stability of the sensor recognition element over time is of great significance for the reliable and long-term operation of a sensor. In the study, responses of the sensor to 5 mM ammonium were monitored during one working day (Fig. 9). It was shown that the sensor signal was highly repeatable (coefficient of variation was about 2.39 %).

A storage stability of the sensor was monitored periodically, and it was observed that on the 116th day after its preparation, the sensor lost more than 90 % of its initial sensitivity.

Finally, the characteristics of the developed sensor were compared with those of the previously reported ammonium-selective electrodes and/or sensors (Table 2). The results obtained in the current study allowed us to place the calixarene-based sensor among other promising variants (either potentiometric or amperometric) known to date. Considering such analytical characteristics as a response time, operational and storage stability, and cost of analysis upon the whole, we may conclude the advantage of the sensors developed here over the existing sensor prototypes for ammonium.

**Table 2:** Comparison of the reported electrodes (sensors) with the proposed ammonium sensor

Recognition element	Transducing mode	Linear range (mol L^−1^)	Dynamic range (mol L^−1^)	Sensitivity	DL (mol L^−1^)	Response time	Storage stability	Ref.
Nonactin in self-plasticizing poly(*n*-butyl acrylate) film	Potentiometric	1 × 10^−5^–1 × 10^−1^	–	59 mV decade^−1^	1.0–4.0 × 10^−6^	–	At least 3 months	[13]
Glutamate oxidase and glutamate dehydrogenase	Amperometric	1 × 10^−5^–3 × 10^−4^	1 × 10^−5^–3.1 × 10^−4^	325.87 nA s^−1^ mM^−1^	2.06 × 10^−6^	10 s	18 days	[14]
PANI/PSSMA (I)	Amperometric	0–1 × 10^−2^	–	2.55 μA mM^−1^	–	–	–	[15]
PANI/PSSMA (II)	Amperometric	0–1.25 × 10^−2^	–	12 μA mM^−1^	–	–	–	
Crown-ether end-capped carbosilane dendrimer (15-crown-5-functionalized carbosilane dendrimer)	Potentiometric	7.60 × 10^−6^–1.0 × 10^−1^	–	53.3 mV decade^−1^	3.9 × 10^−6^	6–10 s	At least 45 days	[16]
Pure culture of *Nitrosomonas sp.*	Amperometric	1.4 × 10^−4^–1.43 × 10^−3^	–	0.030–0.036 n.s.r.	1.4 × 10^−4^	15–25 min (in 5–6 days 10–15 min)	14 days	[17]
SiO_2_/ZrO_2_/phosphate-NH_4_ composite	Potentiometric	1.0 × 10^−6^–1.0 × 10^−2^	7.7 × 10^−7^–4.0 × 10^−2^	31.3 mV decade^−1^	1.58 × 10^−7^	1 min	6 months	[18]
Poly(vinyl chloride) membrane with nonactin	Potentiometric	1.0 × 10^−5^–1.0 × 10^−1^	–	56 mV decade^−1^	8.0 × 10^−6^	20 s	6 months	[19]
Zirconium titanium phosphate ion exchanger	Potentiometric	1.0 × 10^−4^–1	1.2 × 10^−5^–1	42 mV decade^−1^	1.0 × 10^−5^	30 s	Over 2 years (if stored under controlled conditions).	[20]
Copper ion-doped clinoptilolite	Amperometric	2.0 × 10^−5^–1.0 × 10^−3^	2.0 × 10^−5^–1.0 × 10^−2^	191 μA mol^−1^ L	5.0 × 10^−6^	1 min	–	[21]
25,27-di-(5-thio-octyloxy)calix[4]arene-crown-6	Conductometric	1 × 10^−5^–1.5 × 10^−3^	1.0 × 10^−5^–1.0 × 10^−2^	8.22 μs mM^−1^	1.0 × 10^−5^	5–10 s	115 days	This work

*PANI* polyaniline, *PSSMA* poly(styrene sulfonate-*co*-maleic acid) anion, *n.s.r.* normalized signal response, *DL* limit of detection

To study the practical performance of the proposed calixarene-based sensor, it was employed to determine trace amounts of ammonium in spiked water samples. For the analysis, samples from a river, Dnipro located in Kyiv, were filtered then spiked with various concentrations of ammonium and analyzed with the calixarene-based sensor. The recoveries were in the range of 93 to 106 %, indicating good accuracy and validating the potential utility of the present biosensor for ammonium detection in aqueous solutions.

## Conclusions

A novel conductometric sensor for ammonium was elaborated using 25,27-di-(5-thio-octyloxy)calix[4]arene-crown-6 as the selective recognition element, immobilized on the surface of the gold thin-film interdigitated electrodes. It was shown that the immobilization procedure used for the sensor preparation ensured the highly repeatable ammonium sensing with the acceptable sensitivity. The high selectivity of the sensor to ammonium in the media containing widespread interfering ions (potassium, sodium, calcium, magnesium, and aluminum) was observed. Limit of detection of the sensor was 0.01 mM, linear concentration range 0.01–1 mM, and response time 5–10 s. The analytical characteristics of the sensor allowed considering it as an efficient analytical tool for the *on-line* and *on-site* ammonium detection in aqueous solutions.

## Additional file

Additional file 1:
**Method of synthesis of 25,27-di-(5-thio-octyloxy)calix[4]arene-crown-6.** (DOCX 105 kb)

## References

[1] Sawyer J Surface waters: ammonium is not ammonia, Integrated Crop Management News, Iowa State University, 4/21/2008.

[2] Molins-Legua C, Meseguer-Lloret S, Moliner-Martinez Y, Campins-Falco P (2006). A guide for selecting the most appropriate method for ammonium determination in water analysis. Trends Anal Chem.

[3] Katritzky AR Advances in heterocyclic chemistry, Vol. 97., Academic Press, 4/06/2009, 360. http://books.google.com.ua/books?id=G3qNNKYNHM8C&pg=PA220&lpg=PA220&dq=heterocycles.+calixarene&source=bl&ots=8rawI4P9RW&sig=q8GMjIxxScincIureitmRQadOqM&hl=ru&sa=X&ei=xbQiVPzYK6PnygO7nYDQBw&ved=0CC0Q6AEwAg#v=onepage&q=heterocycles.%20calixarene&f=false

[4] Casnati A, Jacopozzi P, Pochini A, Ugozzoli F, Cacciapaglia R, Mandolini L, Ungaro R (1995). Bridged calix[6]arenes in the cone conformation: new receptors for quaternary ammonium cations. Tetrahedron.

[5] Paek K, Ihm H (1996) Synthesis and binding property of tunable upper-rim calix[4]crowns. Chem Letters, 311-312.

[6] Ihm H, Kim H, Paek K (1997). Molecular engineering. Part 2. Influence of side-chain substituents in lariat-type upper rim calix[4]crowns on their binding properties and the reversal of these. J Chem Soc Perkin Trans.

[7] Saiapina OY, Dzyadevych SV, Walcarius A, Jaffrezic N (2012). A novel highly sensitive zeolite-based conductometric microsensor for ammonium determination. Anal Lett.

[8] Dzyadevych SV (2005). Conductometric enzyme biosensors: theory, technology and practice. Biopolym Cell.

[9] Pyeshkova VM, Saiapina OY, Soldatkin OO, Kukla OL, Dzyadevych SV (2008). Enzyme conductometric biosensor for lactose content determination. Biotechnologia Acta.

[10] Bakker E, Pretsch E, Bühlmann P (2000). Selectivity of potentiometric ion sensors. Anal Chem.

[11] Cammann K (1979). Working with ion-selective electrodes.

[12] Dzyadevych SV, Soldatkin AP (2008). Solid-state electrochemical enzyme biosensors. Institute of Molecular Biology and Genetics of National Academy of Sciences. p. 221

[13] Kwan RCH, Hon PYT, Renneberg R (2005). Amperometric determination of ammonium with bienzyme/poly(carbamoyl) sulfonate hydrogel-based biosensor. Sens Actuators B Chem.

[14] Heng LY, Alva S, Ahmad M (2004). Ammonium ion sensor based on photocured and self-plasticising acrylic films for the analysis of sewage. Sens Actuators B Chem.

[15] Luo Y-C, Do J-S (2006). Amperometric ammonium ion sensor based on polyaniline-poly(styrene sulfonate-*co*-maleic acid) composite conducting polymeric electrode. Sens Actuators B Chem.

[16] Chandra S, Buschbeck R, Lang H (2006). A 15-crown-5-functionalized carbosilane dendrimer as ionophore for ammonium selective electrodes. Talanta.

[17] Raud M, Lember E, Jõgi E, Kikas T (2013). *Nitrosomonas sp.* based biosensor for ammonium nitrogen measurement in wastewater. Biotechnol Bioprocess Eng.

[18] Coutinho CFB, Muxel AA, Rocha CG, de Jesus DA, Alfaya RVS, Almeida FAS, Gushikem Y, Alfaya AAS (2007). Ammonium ion sensor based on SiO_2_/ZrO_2_/phosphate-NH_4_ composite for quantification of ammonium ions in natural waters. J Braz Chem Soc.

[19] Schwarz J, Kaden H, Pausch G (2000). Development of miniaturized potentiometric nitrate- and ammonium selective electrodes for applications in water monitoring. Fresenius J Anal Chem.

[20] Hassan SSM, Marei SA, Badr IH, Arida HA (2001). Novel solid-state ammonium ion potentiometric sensor based on zirconium titanium phosphate ion exchanger. Anal Chim Acta.

[21] Walcarius A, Vromman V, Bessiere J (1999). Flow injection indirect amperometric detection of ammonium ions using a clinoptilolite-modified electrode. Sens Actuators B Chem.

