# Supplementary material for: Development of Conductometric Sensor Based on 25,27-Di-(5-thio-octyloxy)calix[4]arene-crown-6 for Determination of Ammonium
Source: Nanoscale Res Lett. 2016 Feb 25;11:105. doi: 10.1186/s11671-016-1317-9 (PMC4766146; doi:10.1186/s11671-016-1317-9)
Supplement: Additional file 1: — Method of synthesis of 25,27-di-(5-thio-octyloxy)calix[4]arene-crown-6. (DOCX 105 kb) [file 11671_2016_1317_MOESM1_ESM.docx]

**Additional file 1. Supplementary data**

**Method of synthesis of 25,27-di-(5-thio-octyloxy)calix[4]arene-crown-6**

The synthesis consisted of three stages.

In the first stage, commercially available tetrahydroxycalyx[4]arene **1** (Scheme A.1) was alkylated by 1,4-dichlorobutane in dimethylformamide (DMF) in the presence of potassium carbonate and a catalytic amount of potassium iodide. As a result, 25,27-di-(4-chlorobutoxy)calix[4]arene **2** (Scheme A.1) in the *cone* conformation was obtained.

Scheme A.1

### The *cone* conformation of compound 2 was confirmed by the presence of [doublet of doublet](http://www.proz.com/kudoz.php/english_to_russian/medical:_instruments/2105038-double_douplet.html)s of spin AB-system of axial (4.26 ppm, 4H, J = 12 Hz) and equatorial (3.39 ppm, 4H, J = 12 Hz) protons of methylene of macrocycle in NMR spectrum.

In the second stage, 25,27-di-(4-chlorobutoxy)calix[4]arene-crown-6 **3** (Scheme A.2) was obtained by the reaction of cyclization of di-(4-chlorobutoxy)calix[4]arene **2** (Scheme A.1) with ditosylatepentaethyleneglycol in the acetonitrile solution in the presence of cesium carbonate. At this stage, due to the template effect of cesium cation the *cone* conformation of compound **2** (Scheme A.1) was transformed into the *1,3-alternate* conformation of compound **3** (Scheme A.2).

Scheme A.2

The final product 25,27-di-(5-thia-octyloxy)calix[4]arene-crown-6 **A** (Scheme A.3) was obtained by the substitution of chlorine atoms in compound **3** (Scheme A.2) by propylsulfide group due to the reaction with propylmercaptan in the tetrahydrofuran solution in the presence of sodium hydride (Scheme A.3).

Scheme A.3

**Synthesis of a compound A**

***Method of obtaining 25,27-di-(4-chlorobutoxy)calix[4]arene 2 (Scheme A.1)***

2.0 g (14.47 mmol L^-1^) of potassium carbonate and 20 mL of dimethylformamide were added to 2.0 g (4.7 mmol L^-1^) of calix[4]arene. The reaction mixture was stirred at room temperature, then 3.5 g (27.6 mmol L^-1^) of 1,4-dichlorobutane and 0.2 g (1.2 mmol L^-1^) of potassium iodide were added. The obtained mixture was kept at 75–80 ° C for 48 h, cooled to room temperature, 100 mL of water were added, extraction with 200 mL of chloroform was performed. The organic layer was washed with 20 mL of 0.5 N hydrochloric acid and 100 mL of water and dried over sodium sulfate. The solvent was removed under reduced pressure. The residue was crystallized from n-butyl alcohol. The precipitate was filtered. The colorless crystalline solid was obtained. Yield 1.32 g (45.6 %). Melting temperature 175–176 °С.

Spectrum NMR ¹Н (СDСl_3_, 300 MHz) δ, ppm: 2.24 (m, 4Н, -C*H_2_*-C*H_2_*-), 3.40 (d, 4Н_equat_*,* Ar-C*H_2_*-Ar), 3.78 (t, 4Н, -C*H_2_*-Cl), 4.03 (t, 8Н, -CH**_2_**-O), ), 4.26 (d, 4Н _axial_, Ar-C*H_2_*-Ar), 6.72 (d. t, 4Н, Ar-H), 6.99 (d. d, 8Н, Ar-H), 8.01 (s, 2Н, ОН). Calculated, %: С 71.39; Н 6.32; Cl 11.70. С_36_Н_38_Сl_2_О_4_. Found, %: С 71.47; Н 6.45; Cl 11.58.

***Method of obtaining 25,27-di-(4-chlorobutoxy)calix[4]arene-crown-6 3 (Scheme A.2)***

50 mL of acetonitrile were added to 1.0 g (1.65 mmol L^-1^) of 25,27-di-(4-chlorobutoxy)calix[4]arene; the reaction mixture was heated to 75–80 °C and 1.2 g (3.68 mmol L^-1^) of cesium carbonate were added. After 30 min, 1.2 g (2.20 mmol L^-1^) of ditosylatepentaethyleneglycol were added. The obtained reaction mixture was kept at 90–95 °C for 48 h, cooled to room temperature; the excess of cesium carbonate and formed cesium tosylate were filtered; the solvent was removed under reduced pressure. 200 ml of chloroform were added, the organic layer was washed with 20 mL of 2 N hydrochloric acid and 50 mL of water, and dried over sodium sulfate. The solvent was removed under reduced pressure. The residue was purified by column chromatography and crystallized from n-butyl alcohol. The colorless crystalline solid was obtained. Yield 0.35 g (43.3 %). Melting temperature 115–116 °С.

Spectrum NMR ¹Н (СDСl_3_, 300MHz) δ, ppm: 1.41 - 1.58 (m, 4Н, -C*H_2_*-C*H****_2_***), 3.58 (t,16Н, ArO-C*H_2_*-CH**_2_** -), 3.38–3.81 (m, 20Н, Ar-O-C*H_2_*-C*H_2_*-O-), 6.84 (d. t (4Н every, $J_{H-H}^{3}$= 7.2 Hz, Ar-H), 7.08–7.11 (d. d (8Н, $J_{H-H}^{3}$= 7.2 Hz, Ar-H). Calculated, %: С 68.39; Н 6.99; Cl 8.78. С_46_Н_56_ Сl_2_О_8_. Found, %: С 70.07; Н 4.86; Cl 8.65.

***Method of obtaining 25,27-di-(5-thia-octyloxy)calix[4]arene-crown-6 A (Scheme A.3)***

10 mL of tetrahydrofuran were added to 0.30 g (1.65 mmol L^-1^) of 25,27-di-(5-thia-octyloxy)calix[4]arene-crown-6; the reaction mixture was cooled to -10 °С; 0.145 g (3.0 mmol L^-1^) of sodium hydride were added. After 30 min, 0.226 g (3.0 mmol L^-1^) of n-propylmercaptan were added; the reaction was kept at 70–75 °С for 6 h, cooled to room temperature; the solvent was removed at reduced pressure. 50 mL of chloroform were added; the organic layer was washed with 20 mL of 1 N hydrochloric acid and 50 mL of water, dried over sodium sulfate. The solvent was removed at reduced pressure. The residual was purified with column chromatography.

Yield 0.16 g (48.6 %). Viscous oil. Spectrum NMR ¹Н (СDСl_3_, 300MHz) δ, ppm: 1.04 (t, 6Н, -C*H_3_*), 1.37–1.45 (m, 4Н, -C*H_2_*-C*H_2_*), 1.61–1.66 (m, 4Н, -C*H_2_*-C*H_2_*-), 2.46–2.54 (m, 8Н, -S-C*H_2_*-), 3.72 (s, 4Н, Ar-O-C*H_2_*-), 3.79 (s, 8Н, Ar-C*H_2_*-Ar), 1.58–1.64 (m, 4Н, -C*H_2_*-C*H_2_***-**), 3.58 (t, 4Н, Ar-O-C*H_2_*-CH_2_ -), 3.38–3.79 (m, 16Н, Ar-O-C*H_2_*-C*H_2_*-O-), 6.82–6.84 (d. t, 4Н, Ar-H), 7.03–7.10 (d. d, 8Н, Ar-H). Calculated, %: С 70.38; Н 7.95; S 7.23. С_52_Н_70_О_8_S_2_. Found, %: С 70.54; Н 8.06; S 7.15.
